# A Methodology and a Web Platform for the Collaborative Development of Context-Aware Systems

**DOI:** 10.3390/s130506032

**Published:** 2013-05-10

**Authors:** David Martín, Diego López-de-Ipiña, Aurkene Alzua-Sorzabal, Carlos Lamsfus, Emilio Torres-Manzanera

**Affiliations:** 1 Centre for Cooperative Research in Tourism, CICtourGUNE, Mikeletegi Pasalekua 71, Donostia-San Sebastián 20009, Spain; E-Mails: aurkenealzua@tourgune.org (A.A.-S.); carloslamsfus@tourgune.org (C.L.); 2 Faculty of Engineering, University of Deusto, Avda. De las Universidades 24, Bilbao 48007, Spain; E-Mail: dipina@deusto.es; 3 Department of Statistics, University of Oviedo, Avda. Luis Moya 261, Gijón 33003, Spain; E-Mail: torres@uniovi.es

**Keywords:** context-aware, toolkit, domain expert, development methodology

## Abstract

Information and services personalization is essential for an optimal user experience. Systems have to be able to acquire data about the user's context, process them in order to identify the user's situation and finally, adapt the functionality of the system to that situation, but the development of context-aware systems is complex. Data coming from distributed and heterogeneous sources have to be acquired, processed and managed. Several programming frameworks have been proposed in order to simplify the development of context-aware systems. These frameworks offer high-level application programming interfaces for programmers that complicate the involvement of domain experts in the development life-cycle. The participation of users that do not have programming skills but are experts in the application domain can speed up and improve the development process of these kinds of systems. Apart from that, there is a lack of methodologies to guide the development process. This article presents as main contributions, the implementation and evaluation of a web platform and a methodology to collaboratively develop context-aware systems by programmers and domain experts.

## 1. Introduction

Ubiquitous Computing is a paradigm whereby all the everyday objects will have an embedded computer and they will be connected to the Internet, providing personalized services to users at any time and place [1]. This vision is a reality in mobile environments. Our smartphones are connected to the Internet and they can provide us with information and services everywhere. But this mobile environment is different from a desktop one. For instance, the screen is smaller, users are on the move and they usually require very specific information at a given time and place. This way, the customization of information is essential for an optimal user experience, especially in these kinds of mobile environments.

Nowadays, there are more than four billion of mobile devices all over the World and it is expected that by the year 2014, there will be more mobile devices connected to the Internet than desktop computers. In addition to this, the 69% of global mobile phone users have a smartphone. These devices are replacing cameras, music players and Global Positioning System (GPS) tools. The 47% of mobile phone users have increased their data use over the year 2011. The 55% of them have reported having downloaded a free mobile application and the 25% have paid for an application. In this manner, the mobile device has become an open window to information and services at any time and place [2]. The challenge is to provide users with personalized information and services while on the move.

One of the key points in order to provide an optimal user experience in ubiquitous computing environments is the context in which the interaction between users and computers is carried out [3]. An example of context information is the location of the user. Location Based Services (LBS) [4] use this context parameter in order to filter the results of a mobile search. In this manner, the information is automatically adapted based on the different context parameters on the move, providing the user with a better search experience.

However, there are several challenges that have to be faced by the scientific community and the industry in order to provide the users with context-aware systems. On the one hand, the development of context-aware systems is not a trivial task for programmers. These systems have to be able to obtain relevant context data in order to identify the situation of users at a given time and place and adapt the behavior of the system to that situation. On the other hand, it can be difficult for programmers to identify and parameterize the situations of the user that the system has to be able to detect, because they do not usually have the needed knowledge about the specific domain where the system has to be deployed. People that can overcome this drawback are domain experts, that is, people that are experts in a specific domain, but not necessarily experts in computer science, who use computer environments to perform their tasks [5]. They can better identify the relevant situations for the system to be developed.

Several software toolkits have been proposed in order to simplify the development of context-aware systems, but there are still some gaps in the reviewed frameworks. On the one hand, not all of them are designed to support users' mobility. This is crucial since users' location is the primary context parameter in context-aware scenarios. On the other hand, the reviewed frameworks offer high-level application programming interfaces for skilled programmers. This makes the involvement of non-technical stakeholders in the development life-cycle, particularly domain experts, almost impossible.

Apart from that, there are no software development methodologies that can be used to guide the implementation life-cycle of context-aware systems.

This article describes a Situation-Driven Development methodology and a context-aware development platform supporting such methodology, called Context Cloud. The aim of the platform is to make the development of context-aware systems easier, even for people that do not have technical skills. This way, the platform provides a web front-end where all the features involved in the development of context-aware systems can be easily configured without coding any programming line.

The methodology has been designed in order to guide the identification and parameterization of the relevant situations that have to be detected by the context-aware system to be developed. The aim of this methodology is to promote the collaboration between programmers and domain experts in the development process. Both the platform and the methodology have been evaluated with real users. The performance of the platform has been evaluated as well.

The article is organized as follows: in Section 2, the related work is presented. In Section 3, the theoretical framework is described. Section 4 presents the development methodology. In Section 5, the development platform is described. Section 6 presents the results and main contributions of this research work. Finally, Section 7 concludes the paper with some brief concluding remarks.

## 2. Related Work

On the one hand, there are several software development methodologies that can be used in order to implement context-aware systems [6] (e.g., waterfall, iterative) but all these methodologies are designed to guide the development process of general software systems. There are other modern approaches like agile methodologies [7] where the final user is involved in the development process. However, the aforementioned methodologies do not consider the specific tasks that are related to the development of context-aware systems. These tasks involve processes such as the context data identification, the context sources selection, the situation parameterization and detection, and the definition of the final system's behaviour once a situation is detected.

Several authors have proposed development methodologies for the implementation of context-aware systems. Henricksen and Indulska [8] proposed a methodology and a modeling language called Context Modeling Language (CML). This language was developed for conceptual modeling of databases that store context data. It has different constructs for capturing the needed classes and context sources, the quality of context data and the dependencies and constraints between context fact types. This way, system designers can specify the requirements of context data needed by the system. The methodology is divided in five different stages: analysis, design, implementation, configuration of the system and validation. Context-oriented Programming (COP) [9] is a programming model that offers mechanisms to adapt the system to be implemented according to the gathered context data. These approaches are focused on system designers and programmers and do not involve non-technical users. In that way, domain experts cannot take part in the development process.

On the other hand, several architectures and frameworks have been proposed in order to support the development of context-aware systems. One of the first implemented approaches is the Context Toolkit [10]. This framework presents an architecture composed of different functional modules in order to acquire, aggregate and interpret context information. It uses key/value pairs in order to model context data. Other approaches like CASS [11] propose a layered middleware architecture that uses a relational data model to represent context data. JCAF [12] is a framework and a runtime environment to develop and deploy contextual computing applications. It uses an object oriented model to represent context data. These three approaches use interpreters to convert acquired raw data into higher level context data, but these transformations cannot be very complex because there is no inference mechanism.

The CoBra [13] middleware proposes a different approach where software agents are used in order to acquire and process context data in a smart meeting room environment. SOCAM [14] and Semantic Spaces [15] are also frameworks based on three different layers, namely a sensing layer, a middleware layer and an application layer.

Mobile frameworks have also been developed in order to create applications that are executed in mobile devices [16]. The main drawback of these frameworks is that they are not powerful enough to support complex context management and reasoning. The number of context parameters used is also low because they are limited to mobile device data sources.

Some authors have proposed visual approaches where domain experts can be involved in the development life cycle. For instance, DiaSuite toolkit [17] comprises a domain-specific design language, a compiler for this language and an editor to define simulation scenarios. The OPEN framework [18] is an ontology-based programming environment for rapid prototyping of context-aware applications. It is based on the configuration of semantic rules in order to trigger predefined actions.

Context Cloud differs from the above toolkits in the following respects. It is designed to promote the collaboration between technical and non-technical users, guided by the designed development methodology. It provides a web environment where context data can be managed using a graphical interface without having to code anything. It also provides geospatial functionalities to manage location context data and all the configurations can be extended at runtime.

## 3. Theoretical Framework

The literature review in the realm of context-aware computing shows that there is no consensus on a definition for the notions of context and situation. The next sections describe the contributions of this research work against the reviewed definitions for these two main concepts.

### 3.1. Context-Aware System

First, let us define a context-aware system. Some authors consider that these kinds of systems are able to adapt their behaviour according to the location of use, the collection of nearby people and objects, as well as changes to those objects over time [19]. Other authors consider that context-awareness is the ability of the computer to sense and act upon information about its environment without explicit user intervention [20,21]. This way, these definitions consider that context-aware systems are reactive systems. In this research work a *context-aware system* is considered as *a reactive hardware or software system that adapts its behaviour to the gathered context data.*

### 3.2. Context

In recent years, there have been several authors that have stated different context definitions. Some of these definitions consider context as the surroundings of the interaction between the user and the application [22]. Other authors consider the activity or the task of the user as the main context information for the system [23]. A third group of authors consider that context is the needed information to characterize the situation of an entity [10].

In this research work, context will be considered from a computing perspective, having into account the third group of definitions mentioned before. This way, *context* is considered as *any information that can be obtained and processed by hardware or software systems, in order to identify the situation of an entity and adapt the system's behaviour to that situation*. Extending the definition of context provided by Dey [10], an *entity* can be a *living being, a place or an object*. The objective of this definition is to provide an operative definition to be applied in the development of hardware and software solutions that use context data in order to adapt their functionalities.

### 3.3. Situation

Apart from the aforementioned context descriptions, there are several definitions for the concept of situation. These definitions have something in common: they consider context as low-level data, while a situation is high-level data. This way, a situation is dependent on the context information and it can be considered as an abstraction of it [24].

In the scope of this work, a situation is defined as the state of the current and past context at a certain region in space and a concrete interval in time that are relevant to identify that situation. There are two main principles that have been taken into account for this definition. The first one is the notion of time. A situation can have temporal boundaries. It also can be related to past or current context according to Allen's temporal logic [25]. The other one is the notion of space, that is, the location where the situation can be identified. For instance, the situation “cooking” could be detected when the user is in the kitchen (space) and it is time to have launch (time). This new definition of situation is more operative for the scope of this research than the ones analyzed in the literature review. Its aim is to facilitate the modeling of situations.

### 3.4. Context and Situation

**Figure 1.**
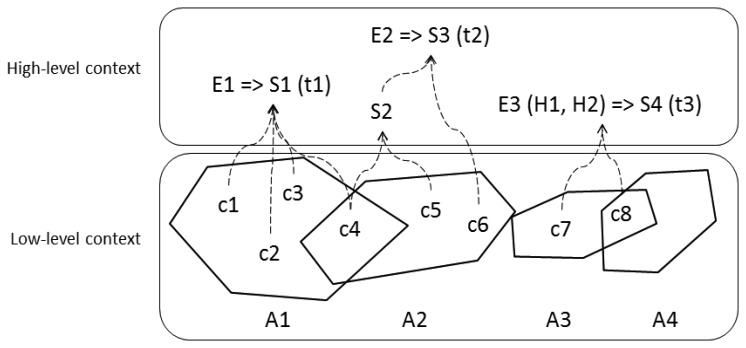
Context and situation.

The previously described concepts of context and situation are graphically explained in the following Figure 1.

Let us imagine that there is a collection of context data (c1, c2, c3, c4, c5, c6, c7, c8). These context data can be obtained and processed by a computing system in order to identify several situations (S1, S2, S3, S4) that are related to different entities (E1, E2, E3).

These context data can be grouped into different subsets, based on different regions in space or areas (A1, A2, A3, A4) where this information is valuable to identify a certain situation at a given interval in time (t1, t2, t3). Also, a situation (S3) can be composed of context data and other situations (S2). A situation (S4) can also be identified using past context data or situations (H1, H2).

## 4. Situation-Driven Development

In order to guide the development process of context-aware systems and involve non-technical domain experts in collaboration with programmers, a methodology has been designed. This methodology considers the different entities' situations that are relevant to adapt the behavior of the system to be developed. Also, it is based on the following premise: programmers have context-aware toolkits that can detect user situations and these toolkits can be configured without programming skills.

Situations are the key element around which all the methodology has been designed. In order to define a situation, five different characteristics derived from the definition of situation have been taken into account. These are related to five different questions that define a situation: the name of the situation (what), the entities that are related to the situation (who), the location where the situation can be detected (where), the time and date range when the situation can be detected (when) and finally, the needed context data in order to detect the situation (how) [26].

The methodology is divided into five different stages: *analysis, configuration, development, validation* and *maintenance*. There are some stages where only the programmer can participate because they require some kind of development.

### 4.1. Analysis

In the analysis stage, domain experts and programmers have to identify all the situations that can be relevant for the system to be developed, specifying a name, a description and the desired behaviour of the system once the situation is detected. Also, each of the identified situations have to be parameterized with the entities that are involved in the situation, the location where the situation can be identified and the interval of time when the situation can be detected. The needed inputs of context data in order to detect the situation have to be specified as well, providing the objective, the conditions and the restrictions for each data type. Finally, the needed outputs once a situation is detected have to be specified. These outputs will be used by programmers in order to adapt the system's behaviour according to the previous specifications. In order to support the analysis stage, a spreadsheet has been designed, where domain experts and programmers can discuss about the needed parameters of each of the identified situations.

### 4.2. Configuration

Once the analysis stage is finished, the toolkit has to be configured with the specified parameters. In this stage, the programmer has to identify and configure the context sources that can provide the defined inputs of data, and configure or implement the providers that are going to obtain these data from the identified context sources. The next step is the configuration of the areas where the situations could be detected, the context data model that will store context data, the mappings between the obtained data and the model, and the inference mechanisms in order to detect the needed situations. These configurations should be done by domain experts with the collaboration of programmers, so the toolkit has to provide configuration mechanisms in order to avoid the usage of programming languages.

### 4.3. Development

In the development stage, programmers have to implement the defined behaviours of the system, processing the high-level outputs generated by the toolkit.

### 4.4. Validation

**Figure 2.**
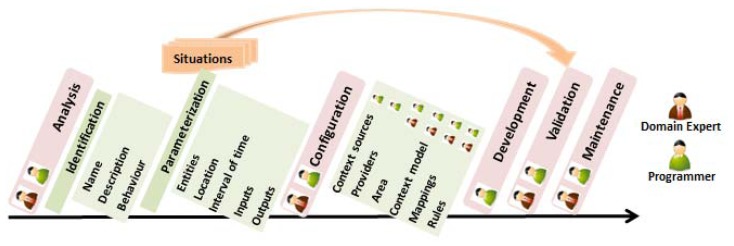
Situation-Driven Development.

The system has to be tested and validated by domain experts and programmers. Also, the final user could be involved in this stage. As shown in Figure 2, each situation has to be parameterized, the toolkit has to be configured and the system has to be implemented and validated. This way, the system is developed using an incremental approach, which has been inspired in agile development methodologies like Scrum or XP [7].

### 4.5. Maintenance

The final stage is the maintenance of the implemented system and the configurations of the toolkit. The maintenance of the system has to be carried out by the programmer and the maintenance of the toolkit should be also carried out by domain experts.

## 5. Context Cloud

This section describes the implemented platform for the development of context-aware systems. The platform has been designed in order to overcome the drawbacks identified in these kinds of toolkits. The previously described development methodology can be used with the platform as well. Next sections present the followed design guidelines, the architecture and the main functionalities of the platform. These functionalities are compared with the reviewed context-aware toolkits.

### 5.1. Design Guidelines

In order to design the platform, several requirements have been identified, which can be considered as the foundations for a platform to develop context-aware systems by domain experts.

#### 5.1.1. Data Model

In order to store the received context information a context data model is needed. This way, the platform can manage data more effectively. There are several data models that can be used in order to manage context information [27], but these have to fulfill the following requirements [28]:

- *Heterogeneity.* The context data model has to represent data coming from very different data sources, which usually provide information in a heterogeneous way.
- *Dependencies and properties.* The relationships between different context entities and their properties have to be modeled.
- *Inference.* Reasoning engines or inference systems have to be applied over the specified data model. This way, high-level information (situations) can be detected.
- *Flexibility.* The defined model has to be flexible enough in order to be extended at runtime. This way, the highly dynamic requirements of these kinds of systems can be supported.
- *Spatial representation.* Location is one of the main context parameters. The model has to provide mechanisms in order to manage this information. Apart from that the model has to have functionalities in order to manage areas or regions in space where the situations can be identified.
- *Time.* The temporal boundaries when a situation can be detected are also relevant for the context model.

#### 5.1.2. Reasoning

As mentioned before, a reasoning engine is needed in order to infer high-level context and to detect situations of entities. The reasoning engine has to be powered by logic rules. This way, domain experts can specify the needed context data conditions to detect situations. Two of the main requirements for the reasoning engine are spatial and temporal reasoning support. Spatial reasoning is needed in order to trigger rules attached to regions in space where situations can be detected once an entity is located inside one of these regions. Temporal reasoning is also needed in order to support Allen's temporal logic [24].

#### 5.1.3. Automatic Context Data Life-Cycle Management

The management of context data involves several tasks. Data has to be transformed into the defined data model, the instances of the model have to be inserted or updated in the knowledge base and data coming from different sources have to be aggregated if they are related to the same entity instance. This data management can be quite repetitive and mechanisms that can provide automations are needed.

#### 5.1.4. Extensibility

The platform needs to be flexible enough in order to be extended at runtime [29]. In such dynamic environments new context sources could be required in order to identify new situations. This way, the platform has to allow the configuration of the data model and the defined rules according to new context data requirements at runtime.

#### 5.1.5. Mobility

Location is the main context parameter to be considered in order to personalize the behaviour of context-aware systems [4]. The entities that can be involved in a certain situation can be on the move (e.g., Person, Car and Device), so the architecture of the platform has to integrate a Geospatial Information System (GIS) in order to manage their location. Also, this GIS service is needed in order to manage the areas where situations can be identified and to detect the entities that are located inside those areas.

#### 5.1.6. Web Development Environment

The platform has to be a web application that can be configured at any time with any connected device. It needs to provide the user with a visual environment in order to manage context data using the user interface [30]. Like that, domain experts can easily access and modify all the configurations in real time without having to install any development environment.

### 5.2. Architecture

The architecture of the platform is based on the common layers identified in the reviewed works [21]: context sources layer, providers layer, data management layer and application layer. Even so, some advances in complementary scientific and technological fields have been identified and applied to improve the architecture of these kinds of toolkits. This way, three main ideas have been taken into account in the design stage of the architecture: the Web of Things [31], Cloud Computing and End-User Programming [32] paradigms.

The Web of Things principles can be applied to the sources layer. This way, context sources must have a web end-point in order to get data from them and to interact with them in a RESTful way. In this manner, these principles can provide a higher level of abstraction over the drivers and low-level mechanisms that these sources expose in order to get data from them.

Cloud Computing has been considered in order to design the context data management layer. This layer can be deployed in any web server and it can be configured using a web user interface. In this manner, the platform can scale according to the requirements of the system. The maintenance of the platform can be externalized as well.

The End-User Programming paradigm has been considered in order to involve domain experts in the development process. The users of these toolkits must have the knowledge about what is happening behind the scenes and they must have the control over the managed context data without having programming skills [33].

**Figure 3.**
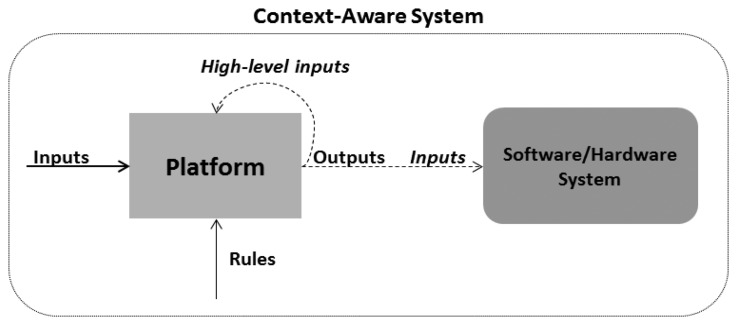
Context data processing pattern.

As previously defined, context-aware systems are considered as reactive systems that adapt their behavior to the detected situations of the entities. This way, the platform has been designed in order to generate the needed high-level outputs that will be used by software or hardware systems in order to have a reactive behaviour. There are several context processing patterns that have been identified in the literature review [34], but they are not applicable to a scenario where domain experts configure the system in order to manage context information and produce high-level outputs. In order to solve this gap, the pattern shown in Figure 3 has been used.

In this pattern, context data is obtained or received by the platform and it produces several outputs with information about the identified situations. Also, the generated outputs can be used as high-level inputs. The outputs (situations) can be modeled using rules that domain experts need to configure based on their knowledge on the application domain.

**Figure 4.**
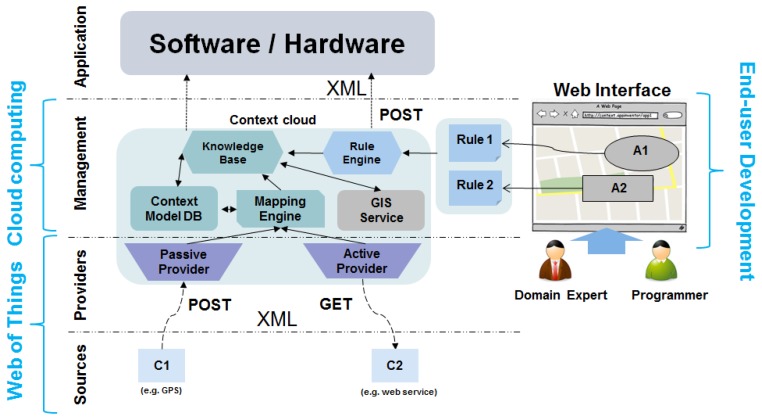
Architecture of Context Cloud.

Figure 4 shows the architecture of the platform, which is divided into four different layers: sources, providers, management and application. This way, Context Cloud can be considered as a black box that receives inputs from the identified context sources and produces outputs triggered by the defined context rules. These rules are used to identify situations according to the obtained context data and the defined data model.

#### 5.2.1. Sources

The first layer is where all the context sources are. These sources are usually distributed and provide heterogeneous data. This is the case of sensor networks, web services, mobile devices or data bases. Situation identification from low-level data provided by these kinds of sources is a challenging task where imperfections of context data source readings and context data uncertainty have to be managed [29].

Sources must be connected to the Internet and they must provide context data in XML or JSON format in order to be processed by the platform. The programmer has to configure the context sources in order to fulfil these requirements. For the scope of this research it is supposed that data coming from the context sources have a reasonable quality.

#### 5.2.2. Providers

Providers are software components that obtain or receive data from the identified context sources. There are two types of providers: passive and active providers. The passive ones, wait until any context source proactively sends an HTTP POST request with the needed context data. Active providers can be configured in order to obtain context data making periodical HTTP GET requests to the source. The interactions between the platform and the context sources are made in a RESTful way, following the best practices established by the community of the Web of Things.

#### 5.2.3. Management

The management layer is composed of several modules. The Context Model Data Base can store a context data model based on context entities (e.g., Person, City, Museum) and properties (e.g., name, temperature) that are internally modeled using Java Bean classes. This data model will be populated by the received context data and transformed into events that are going to be saved (or updated) in the Knowledge Base.

The transformation between data coming from the sources and the data model is performed by the Mapping Engine, according to the user's defined mappings. Every data that is received and transformed into an instance of an entity is automatically considered as a new event for the system, providing a timestamp to it. This way, rules can use temporal operators (e.g., after, before) in order to manage these events.

The GIS component is used to store the created areas where situations can be identified. It also translates location coordinates of context entities into registered area names. The platform provides a context history of the last ten areas where an entity has been detected.

The Rule Engine validates and stores the Event Condition Action (ECA) rules [35] that are created in order to identify the needed situations. It is also the responsible for firing all the rules against the instances of entities that are stored in the Knowledge Base. Every time a new rule is created, it has to be linked to an existing area. This way, the rules that are defined for a certain area, will only be fired if any of the context entities that are specified in the rule condition are located in that area.

Thus, situations can be defined based on specific areas and the context data that is relevant to these concrete areas. Furthermore, rules can produce outputs using HTTP POST requests in order to send data about the detected situation in XML format.

The platform can be configured using the web front-end where programmers and domain experts can create areas, rules, context entities, mappings between context sources and entities and they can configure the context data gathering process from the identified context sources by the use of dialogs.

#### 5.2.4. Application

Finally, the upper layer is the application layer. Here is where all the systems that use the produced high-level outputs are. These systems can use the generated outputs in order to react to the detected situations and personalize the behavior of the system.

### 5.3. Configuration of the Platform

**Figure 5.**
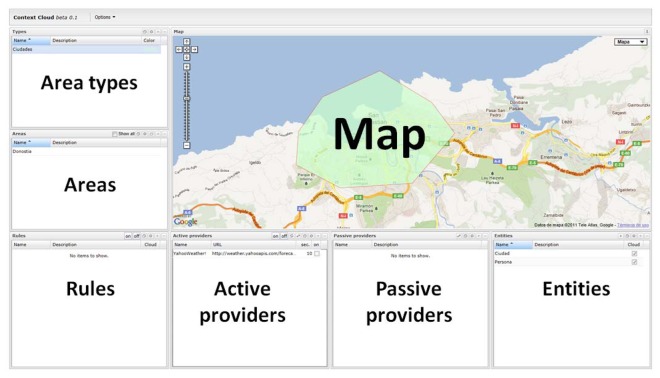
Configuration panel.

Context Cloud has different features that can be configured using the web interface. As shown in Figure 5, this interface is divided into different widgets that show information about the configured items: providers, context model, mappings, areas and rules.

#### 5.3.1. Providers

Context data acquisition from external context sources can be configured using the dialogs that are available to create providers. Active providers can be configured in order to make periodic GET requests to the selected context sources and get a document with the context data in response. Also, passive providers can be configured. These providers can receive XML or JSON documents.

More than one provider can be used in order to get data for the same context entity type. This facilitates the composition of context data coming from different sources. For instance, let us imagine that two types of location data sources are being used, one based on WiFi (indoors) and the other one based on GPS (outdoors). Then, two different providers could be configured in the platform in order to obtain the location coordinates from these sources.

After that, the mappings for these two providers could be configured in order to store data in the location properties of the same context entity type. Rules could be configured in order to prioritize location data based on the quality of the source.

#### 5.3.2. Context Model

**Figure 6.**
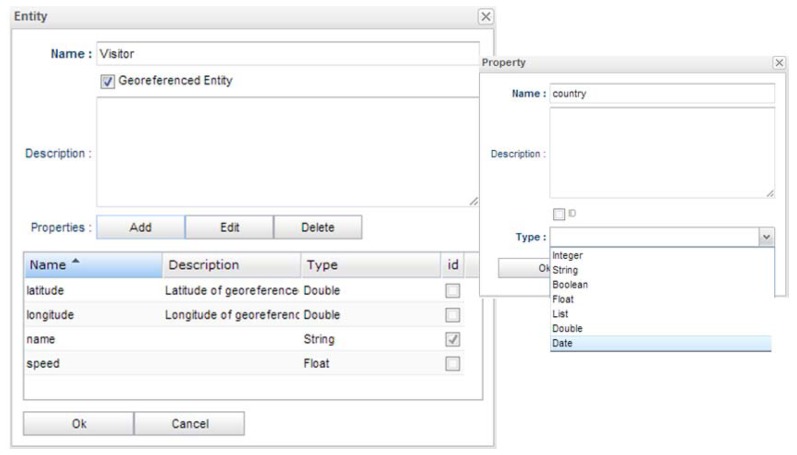
Entity creation dialog.

The platform allows the creation of a context model defining entities with different typed properties. Figure 6 shows the context entity creation dialog.

The entity name, the description and the properties can be created. Every entity must have an identifier property in order to aggregate data coming from different sources that are related to the same entity instance.

An entity can also be marked as a geo-referenced entity, which means that it will contain latitude and longitude coordinates that are going to be managed by the platform's GIS component. For instance, these data coordinates could be received by a passive provider from a GPS device (context source) in order to be stored in the Visitor's latitude and longitude properties using a suitable data mapping configuration.

#### 5.3.3. Mappings

**Figure 7.**
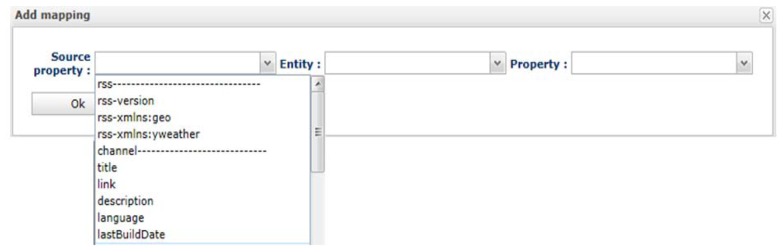
Mapping configuration.

As aforementioned, in the mapping process the obtained context data can be mapped to context entity properties. Context data is interpreted by the platform using the elements, properties and values of the XML or JSON documents coming from the sources. For instance, Figure 7 shows a mapping dialog with all the element s and property names that have been obtained from an XML document. To configure a new mapping, the context source data, the entity and the property of that entity have to be specified.

#### 5.3.4. Areas

The platform also provides widgets in order to create areas defined by polygons over a Google Maps layer. The created areas can be grouped into area types as well.

#### 5.3.5. Rules

**Figure 8.**
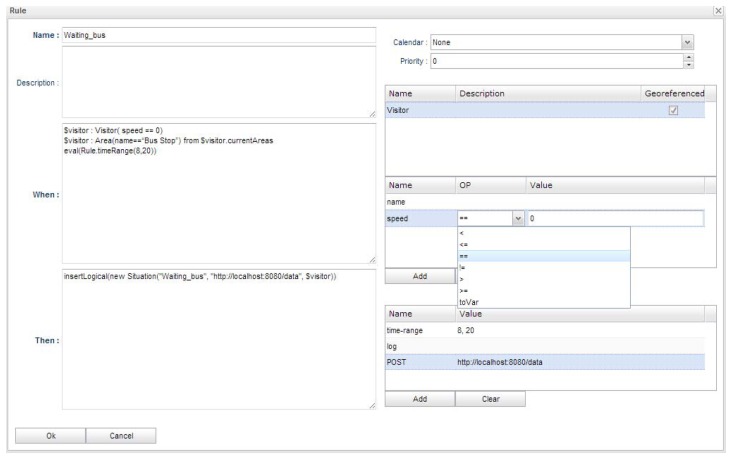
Rule creation dialog.

The rule creation dialog allows the configuration of the needed conditions in order to detect the situations. To create a rule, it must be assigned to an area. Figure 8 shows the dialog to create rules. A name and a description for the rule have to be provided. The dialog also has two text areas where the rule conditions (when) and the rule actions (then) can be specified. These text areas can be edited programmatically specifying the code for the rule or they can be edited using the controls on the right side of the dialog. These controls will automatically translate the configurations into rules.

There are several controls that can be used in order to configure a rule. A calendar option is available, where a date range can be specified for the rule. Also, the priority of the rule can be specified, the higher the number, the higher the priority. The dialog shows all the available entities, their properties and the conditions that can be applied to these properties.

Also, some useful functions are listed. The “log” functionality can be used to print a message in the debugging console of the platform. The “POST” function can be included in the consequence of a rule in order to trigger a POST request to any external web service end-point.

The rule that is shown in the example of Figure 8 has been created in order to detect that a visitor is waiting for the bus. The situation can be detected when a visitor is inside a bus stop. Other relevant context data are the visitor's location, speed, date and time. This way, there is one primary context source that has been used, the visitor's mobile device.

The mobile device has been configured in order to send location coordinates and speed data from the embedded GPS sensor to a passive provider that has been configured using the platform. The context model is composed of a geo-referenced entity, “Visitor”, with some properties: name (id), latitude, longitude and speed. The mappings have been configured and an area has been created around the bus stop named “Bus Stop”.

This way, the configuration of the rule creates the following lines of code:

$visitor: Visitor(speed == 0)
$visitor: Area(name==“Bus Stop”) from $ visitor.currentAreas
eval(Rule.timeRange(8,20))

Several conditions have been defined. A date range (weekday) has been specified using the calendar control. Also a time range has been specified using the function “time-range”. The first condition of the rule indicates that the speed of the visitor must be zero.

The second condition has been automatically generated by the platform because the rule has been created in order to be linked to the “Bus Stop” area. This condition is necessary in order to trigger the rule only when the visitor is at the defined “Bus Stop” area.

The consequence of the rule has been configured with the “POST” function. In this case, if the rule is triggered, it will send information about the Visitor entity instance to the specified web end-point.

**Figure 9.**
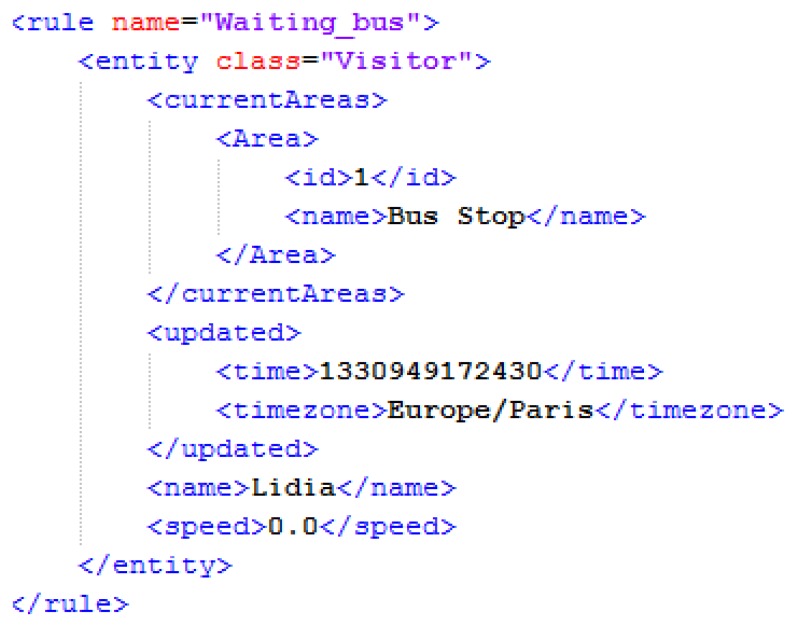
XML output.

An example of the XML document that the rule will send once the situation is detected is shown in Figure 9. This document contains information about the Visitor entity instance, including all its context data properties and values, and the information of the area where the situation was detected.

The name of the rule indicates the name of the situation that has been detected. Also, a timestamp element is included with the last update time of the entity instance.

### 5.4. Discussion

Some functionalities that would be needed in order to fulfil the presented theoretical framework and to support the designed development methodology have been analysed over the reviewed frameworks.

As shown in Table 1, Context Cloud presents more functionalities than the rest of the reviewed toolkits. For instance, it is the only one that automatically manages context data life cycle (a). This management involves the automatic conversion from gathered raw context data to the context data model and automatic updates of current context data and storage of past context data. It also deletes context entities instances from the knowledge base when a registered context source is unregistered or it is no longer available. It is extensible at runtime (b), that is, the user can update the context model, the rules and the areas whenever it is needed. Also, a geographic information system is included in order to support user's mobility (c). Visual programming is supported (d) and the platform can be configured using a web front-end (e), which makes it accessible from any Internet connected device. The platform is suitable not only for programmers (f) but also for domain experts (g).

## 6. Evaluation

The development methodology and the platform have been evaluated with real users. Apart from that, the performance of the reasoning engine of the platform has been evaluated as well.

### 6.1. Reasoning Performance

The performance of the reasoning process in rule production systems may vary depending on several factors and it can be a bottleneck for the rest of the system [36]. In order to evaluate the performance of Context Cloud, and more precisely the reasoning performance, some tests have been carried out in a controlled environment. As a reference, a maximum reasoning time of a second has been considered in order to not affect the final user experience [37]. The following parameters have been configured in the tests.

- The number of context entity instances, with a minimum of a thousand instances and a maximum of 20 thousand instances.
- The context entity properties, where the number of properties per entity vary between one and five.
- The number of rules, between a hundred rules and five hundred rules.
- The number of entities used in the rule conditions, with one or two entities.

It has been used a laptop with 4 GB of RAM and 1 GB of memory reserved for the Java Virtual Machine. The results of the designed tests are the following.

- The higher the number of entity instances in the knowledge base, the worst the performance of the reasoning process.
- The higher the number of entity properties used in the rule conditions the better the performance of the reasoning process.
- The higher the number of rules, the worst the performance of the reasoning process.
- The higher the number of entities used in the conditions of the rules, the worst the performance of the reasoning process.

The conclusions of the reasoning performance tests are that the number of rules and the number of instances should be minimized in order to have a good performance, while the number of properties used in the rule conditions and the available RAM memory should be maximized. If this considerations or advices are not enough, the platform could be configured in order to be deployed in a server cluster because of the cloud nature of the platform. This way, the reasoning process could be distributed between different server instances.

### 6.2. User Evaluation

The platform has been validated with 20 participants. The designed evaluation has been inspired in a tourism scenario [38,39], so the non-technical users were experts in the tourism domain.

The participants carried out the evaluation in pairs composed by a tourism domain expert and a programmer. A tourism expert has been considered as a person whose work is related to the tourism industry or whose education is focused on social sciences. Due to the specific technical skills and professional profiles that have been required in order to carry out the evaluation tests, the sample of participants has been reduced. However, this sample can be considered as a sufficient sample according to the results that are explained in next sections.

#### 6.2.1. Methodology

First of all, the users were introduced to the Context Cloud platform and to the experiment's objectives. They were instructed on how to configure the platform and they were given an example on how to identify a situation using the methodology. The participants were given a text document where four different situations were described.

- Waiting for the bus (S1): the visitor waits for the bus at Bus Stop A and she receives an SMS with the estimated time of arrival for the next bus.
- Sunbathing (S2): the visitor is at the beach when she receives an SMS advising her that she should use sun cream because the temperature is higher than 30 °C.
- Waiting for the bus (S3): the visitor waits for the bus at Bus Stop B and she receives an SMS with the estimated time of arrival for the next bus.
- Arriving to the hotel room (S4): the air conditioning is activated when the visitor goes into the room.

The situations were simulated using the Siafu Context Simulator [40]. It was configured to send context data about the visitor on the move to the platform. The simulator also provided some web services in order to obtain the weather information and to access the air conditioning system of a simulated Hotel. The participants had to configure the platform in order to detect the above mentioned situations. During the test, an external observer annotated all the problems that the participants found using the platform. Also, once a situation was detected, the time spent in its configuration was annotated.

After having completed the user experience, each participant had to fill out a questionnaire based on a six-level likert scale, with values from 1 (totally disagree) to 6 (totally agree). The used survey was designed on the Technology Acceptance Model (TAM) literature, and in particular, it was adapted from David's studies [41]. This way, three constructs were considered: Perceived Ease of Use (PEOU), the Perceived Usefulness (PU), and the Behavioral Intention (BI).

#### 6.2.2. Results

95% of the participants find that learning the methodology is easy and the 100% of them state that it eases the collaborative work. Also, the 100% of the participants find that the methodology is useful to work with the platform, and that it is useful to develop context-aware systems.

The 95% of the participants find that learning how to use the platform is easy. The 75% of the participants find it easy to get Context Cloud to do what they want to do. However, the other 25% disagree on that. The reason is that the participants were not used to work with these kinds of toolkits. The 95% of the participants also find that the interaction with Context Cloud is clear and understandable. The 90% of the non-programmers state that it would be easy for them to become skillful at using the platform.

The perceived utility of the platform is also highly supported by domain experts. The 100% of the domain experts state that using Context Cloud in their jobs would enable them to develop context-aware systems more quickly and that it would make it easier to develop context-aware systems. Also, all of the participants would recommend other users to use the platform and they would use it in future developments. In addition to this, the 80% of them would pay for the system.

The average time spent by each of the pairs to solve the evaluation test was 89 min. It is relevant that the they spent an average time of 37 min in order to solve the situation number one, while for the rest of the situations, the average time was 17 min. This means that once they know how to configure the platform in order to identify the first situation, it is easier for them to configure it for the rest of the situations. This way, the learning curve is steep, that is, the participants learn in a very short period of time how to use the platform successfully.

During the user evaluation, the external observer realized that the excel sheet promotes the discussion between domain experts and programmers during the analysis stage of the methodology. In the configuration stage, some of the non-programmers used the platform in order to configure some of the situations. More precisely, 12 out of 40 situations were totally configured by domain experts. The most difficult part of the learning process was to identify the needed context entities to create the context model. Also, they had problems figuring out which were the needed outputs that the platform needed to produce.

## 7. Conclusions

This research work describes a situation-driven development methodology designed to promote the collaboration between programmers and domain experts in the implementation of context-aware systems. In addition, a platform to ease the development of context-aware systems has been implemented. This platform supports the designed methodology and it is particularly thought for non-technical users. Both the methodology and the platform have been successfully evaluated and validated with real users.

Thanks to the designed platform, users with no programming skills can actively participate in the development life-cycle of context-aware services. This way, programmers can focus on the interactions between the platform and the context sources, and the management of all the outputs that are triggered by the rules in order to develop all the business logic of the service to be implemented.

The results of the user evaluation evidence that the platform can be used in order to implement context-aware systems. It is clear that programmers become skillful users of the platform easier than domain experts, but these also state that they could be skillful at using the platform easily as well. Furthermore, these results evidence that domain experts can be involved in the configuration of the platform, and thus, in the development process guided by the designed methodology.

The development methodology, the platform, and the results obtained from the user evaluation have a fundamental implication in the evolution of Context-Aware Computing. The involvement of domain-experts in the development process of context-aware systems is essential, which implies that the methodologies and tools used to implement such systems have to be adapted to people that do not have programming skills. This has a direct impact on both the design of the processes that have to be taken into account in the development of context-aware systems and the design of toolkit architectures that support the development of such systems. Only domain-expert and end-user involvement on the design and implementation of context-aware systems can ensure that those actually address the end-user real needs.

As future work, the enhancement of the reasoning process is planned. This includes the study of complementary reasoning algorithms based on Naïve Bayes classifiers, Bayesian networks or Hidden Markov Models. There is also a degree of uncertainty in the reasoning process that has not been considered in the implementation of platform. Being able to measure the probability with which a certain situation may happen will be considered in future iterations of the development of the platform.

## Figures and Tables

**Table 1.** Comparison of context-aware development toolkits.

	**a**	**b**	**c**	**d**	**e**	**f**	**g**
**Context Toolkit**	-	-	-	-	-	**x**	-
**JCAF**	-	-	-	-	-	**x**	-
**CASS**	-	-	-	-	-	**x**	-
**SOCAM**	-	-	-	-	-	**x**	-
**CoBra**	-	-	-	-	-	**x**	-
**Semantic Spaces**	-	-	-	-	-	**x**	-
**DiaSuite**	-	-	-	**x**	-	**x**	**x**
**OPEN**	-	-	-	**x**	**x**	**x**	**x**
**Context Cloud**	**x**	**x**	**x**	**x**	**x**	**x**	**x**
